# Habitat-Associated Phylogenetic Community Patterns of Microbial Ammonia Oxidizers

**DOI:** 10.1371/journal.pone.0047330

**Published:** 2012-10-09

**Authors:** Antoni Fernàndez-Guerra, Emilio O. Casamayor

**Affiliations:** Biogeodynamics&Biodiversity Group, Centre d'Estudis Avançats de Blanes CEAB–CSIC, Blanes, Spain; University of Waterloo, Canada

## Abstract

Microorganisms mediating ammonia oxidation play a fundamental role in the connection between biological nitrogen fixation and anaerobic nitrogen losses. Bacteria and Archaea ammonia oxidizers (AOB and AOA, respectively) have colonized similar habitats worldwide. Ammonia oxidation is the rate-limiting step in nitrification, and the ammonia monooxygenase (Amo) is the key enzyme involved. The molecular ecology of this process has been extensively explored by surveying the gene of the subunit A of the Amo (*amoA* gene). In the present study, we explored the phylogenetic community ecology of AOB and AOA, analyzing 5776 *amoA* gene sequences from >300 isolation sources, and clustering habitats by environmental ontologies. As a whole, phylogenetic richness was larger in AOA than in AOB, and sediments contained the highest phylogenetic richness whereas marine plankton the lowest. We also observed that freshwater ammonia oxidizers were phylogenetically richer than their marine counterparts. AOA communities were more dissimilar to each other than those of AOB, and consistent monophyletic lineages were observed for sediments, soils, and marine plankton in AOA but not in AOB. The diversification patterns showed a more constant cladogenesis through time for AOB whereas AOA apparently experienced two fast diversification events separated by a long steady-state episode. The diversification rate (γ statistic) for most of the habitats indicated γ_AOA_ > γ_AOB_. Soil and sediment experienced earlier bursts of diversification whereas habitats usually eutrophic and rich in ammonium such as wastewater and sludge showed accelerated diversification rates towards the present. Overall, this work shows for the first time a global picture of the phylogenetic community structure of both AOB and AOA assemblages following the strictest analytical standards, and provides an ecological view on the differential evolutionary paths experienced by widespread ammonia-oxidizing microorganisms. The emerged picture of AOB and AOA distribution in different habitats provides a new view to understand the ecophysiology of ammonia oxidizers on Earth.

## Introduction

Microbial nitrogen transformations modulate the rate of key ecosystem processes, such as primary production and decomposition [1]. Ammonia oxidation, the first step of nitrification, is a biogeochemical process of global importance in natural and artificial ecosystems worldwide. For many years, ecologists remained perplexed with the nitrifying capacity of many ecosystems where apparently ammonia oxidizers were far below detection limits, particularly under the most oligotrophic conditions [2]. Microbial ammonia oxidation was initially considered to be restricted to a few bacteria, specifically within the phylum Proteobacteria, which under laboratory conditions mostly show an affinity threshold for ammonium higher than the concentrations usually found *in situ* [3]. Metagenomic studies carried out a few years ago in seawater [4] and soil [5] showed a different *amoA* gene related to the phylum Thaumarchaeota, and the presence of the *amoA* gene in widespread Archaea and different habitats has been widely detected since then.

In marine environments, for instance, nitrification accounts for about half of the nitrate consumed by growing phytoplankton at the global scale [6] and is responsible for the deep ocean nitrate reservoir [7], the largest pool of reactive nitrogen in the biosphere [8], [9]. In soils, the ammonia-oxidizing archaea (AOA) apparently dominate over ammonia-oxidizing bacteria (AOB) [9]. However, ammonium concentrations in many soils have increased in recent years as a result of both land-use changes and increases in atmospheric ammonium concentrations [10], and this may influence the microbial ecology of the nitrification process [11]. Finally, nitrification could remove excessive ammonium nitrogen and prevent lakes from eutrophication [12], and ammonia oxidizers adapted to life in sludge and bioreactors can efficiently help to remove excess of nitrogen [13], [14].

Ammonia oxidizers play a fundamental role in the connection between biological N fixation and anaerobic N losses, and are widely detected in a large variety of aquatic and terrestrial environments [15]–[16]. Both AOB and AOA have colonized similar worldwide distributed environments but with different degrees of success in abundance, activity, and distribution [11], [17]–[20]. The molecular ecology of the ammonia oxidation process has been extensively explored surveying the subunit A of the Amo [19], [22]–[26]. Both AOA and AOB contain the amoA gene encoding the alpha subunit of the Amo; however, the gene sequence has evolved separately in each of the phylogenetically distinct but physiologically related ammonia oxidizers microorganisms [5]. In the present study, we explored the phylogenetic community ecology of AOB and AOA assemblages, analyzing differences in composition and richness among environments, detecting habitat-phylogeny associations, and unveiling the historical context of the evolutionary events (cladogenesis) captured in the reconstructed *amoA* gene phylogeny.

## Results

Ammonia oxidizers were detected in 11 different habitats worldwide, including soil, sediment, marine plankton, freshwater plankton, organisms-associated, wastewater, sludge, biofilm, hot spring, hydrothermal vent, and the “cultured” habitat (according to the habitat annotation based on the EnvO-Lite description as the microbial assemblages which develop in bioreactors and biofilters). As mentioned in Methods, we assigned the microbial strains isolated in the laboratory to their original habitats. Bacterial and archaeal ammonia oxidizers shared most of these habitats with a few exceptions: AOB were absent in the database for hot springs and hydrothermal vents, whereas the low number of sludge and biofilms AOA sequences recovered from GenBank did not fit the minimal number of sequences required to be included in our analysis. Recent research in these two habitats [14], [20]–[21] is increasing the number of sequences available for future meta-analyses.

**Figure 1:**
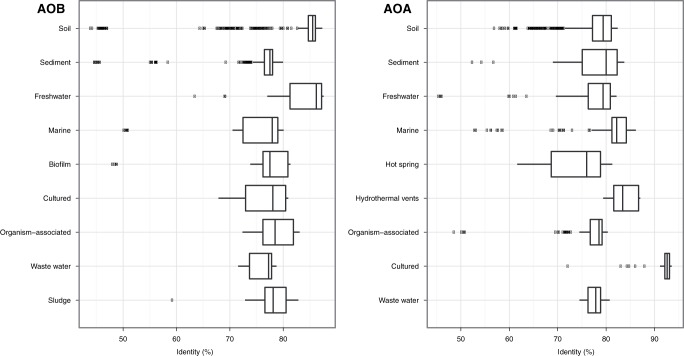
Level of *amoA* gene sequence divergence detected in each habitat for bacterial (left panel) and archaeal (right panel) ammonia oxidizers. The boxplot represents all-against-all pairwise alignment identities for the gene sequences compiled from each habitat and for each domain.

To examine the sequence divergence present in each habitat we carried out an all-against-all pairwise alignment (Fig. 1). We noticed two trends in the *amoA* gene dataset. First, a median value between 75 and 85% identity was detected in essentially all the natural habitats explored. Second, a substantial number of low identity sequences (below 60% identity) were present in several of the habitats investigated. We explored these outliers to rule out cross-contamination among habitats and found they were specific sequences from each habitat.

**Figure 2:**
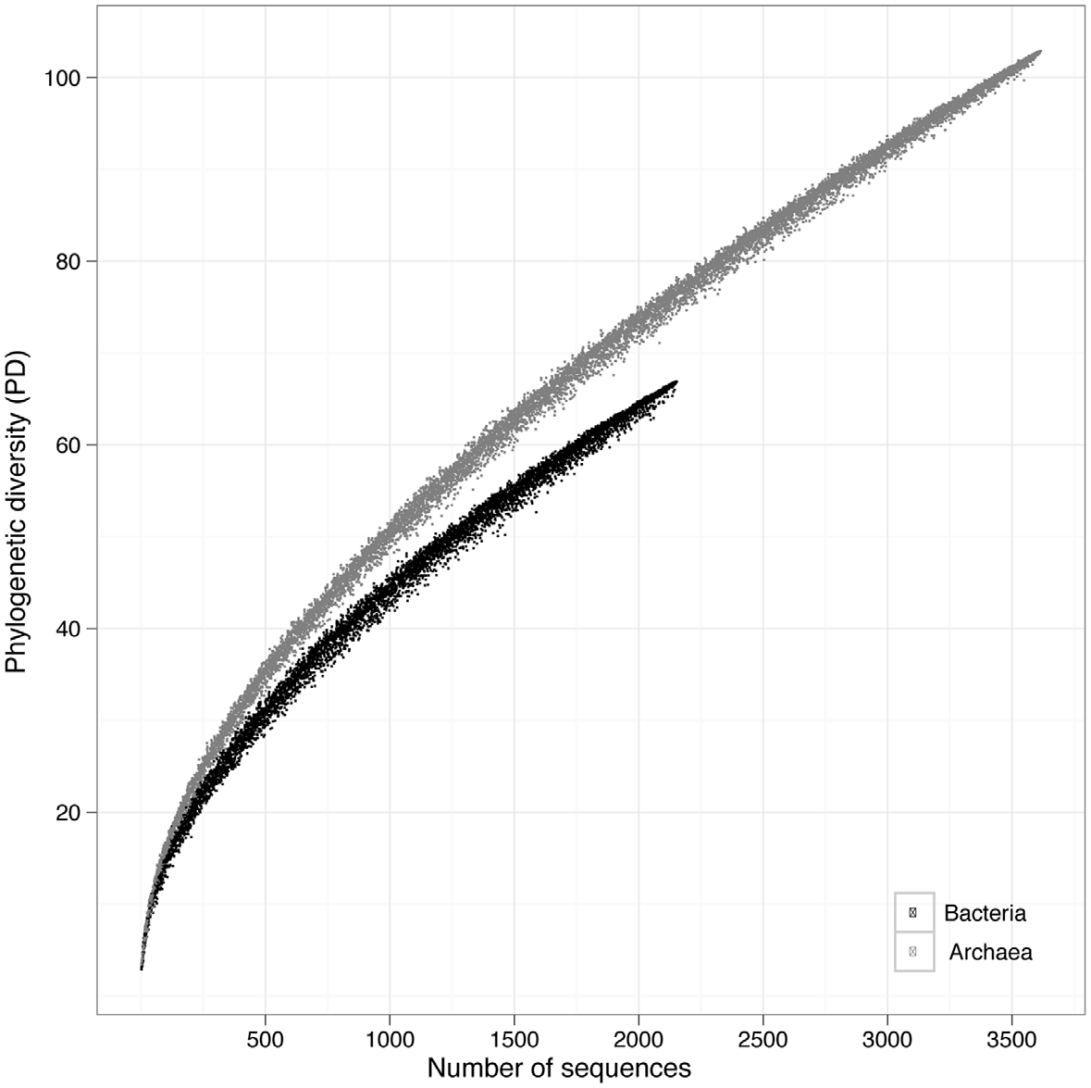
Phylogenetic diversity rarefaction curves for the whole dataset of bacterial and archaeal *amoA* gene sequences. Larger phylogenetic richness is observed in AOA than in AOB for an equivalent sampling effort.

**Figure 3:**
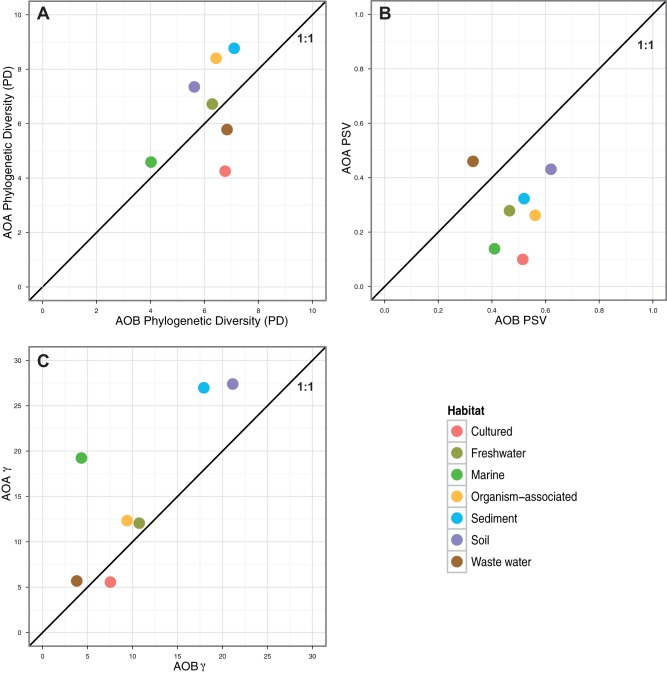
Scatter-plot comparing the AOB vs. AOA. A) phylogenetic diversity (PD), B) phylogenetic species variability (PSV) and C) diversification rates (γ-statistic) for the different shared habitats. See Table S1 for data.

To statistically analyze the phylogenetic richness distribution within each community we calculated the phylogenetic diversity (PD) index from the maximum-likelihood inferred trees after correction for unequal sample size (Table S1). Overall, the PD rarefaction curves consistently showed larger phylogenetic richness in AOA than in AOB for an equivalent sampling effort (Fig. 2). Neither AOA nor AOB reached the plateau for the PD accumulation, indicating that the currently known phylogenetic richness of the *amoA* gene is far from being fully discovered. The PD values for the AOA and AOB shared habitats (Fig. 3A) showed sediments containing the highest phylogenetic richness and marine plankton the lowest. AOA were phylogenetically richer than AOB in both plant- and animal-associated (organism-associated) habitats, and soil. The opposite was found in bioreactors (“cultured” habitat) and wastewater. Interestingly, freshwater AOA and AOB were phylogenetically more diverse than their marine counterparts. AOA in hydrothermal vents and AOB in biofilms showed the lowest PD (Table S1). For most of the explored habitats, AOA were generally more diverse than AOB. Interestingly, the phylogenetic structure captured by the phylogenetic species variability index (PSV) showed AOB to be phylogenetically more overdispersed than AOA in most of the habitats but wastewater (Fig. 3B). A general view on the reconstructed tree topologies showed inconsistent phylogenetic clustering for the AOB recovered from the same type of habitats, in agreement with the picture captured by the PSV index. Conversely, three large phylogenetic clusters were found for AOA, i.e., sediment, soil, and marine plankton.

**Figure 4:**
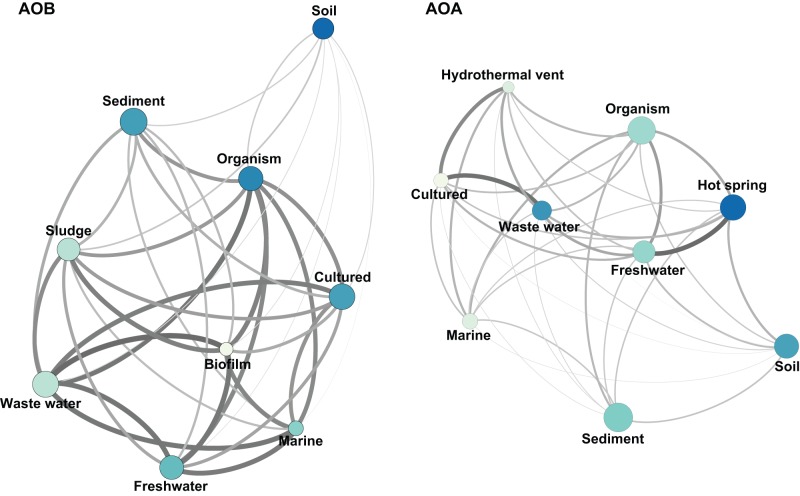
Graphical representation of AOB and AOA Unifrac distance matrices. A thicker line represents phylogenetically more similar communities. Phylogenetic diversity (PD) represented as node size, large nodes have larger PD values. Phylogenetic species variability (PSV) represented as node color, darker nodes have larger PSV values. The length of the edges does not contain information.

The habitat-phylogeny associations were numerically analyzed with UniFrac distances (UD). The UniFrac matrices were represented graphically for AOB and AOA (Fig. 4) to visualize in a quantitative way the community dissimilarity among different habitats. The AOB soil community showed the weakest connections with the remaining nodes in the UD graph, indicating the most distant phylogenetic relatedness (Fig. 4), followed by AOB present in sediments. The remaining habitats showed a closer community structure among the different AOB assemblages. Interestingly, the bacterial *amoA* genes found in wastewater were the closest related to the remaining habitats (the strongest connections with the remaining nodes in Fig. 4). Conversely, the AOA communities as a whole were more dissimilar to each other, showing weaker interlinks (Fig. 4). For AOA, the strongest relationships were observed between freshwater and hot springs assemblages, as well as between wastewater and bioreactors. Soil, sediment and marine plankton were more unrelated to each other and to the remaining assemblages. Overall, AOB were phylogenetically more closely related among habitats than the archaeal counterparts. In turn, AOA were more phylogenetically clustered by habitat than bacteria.

**Figure 5:**
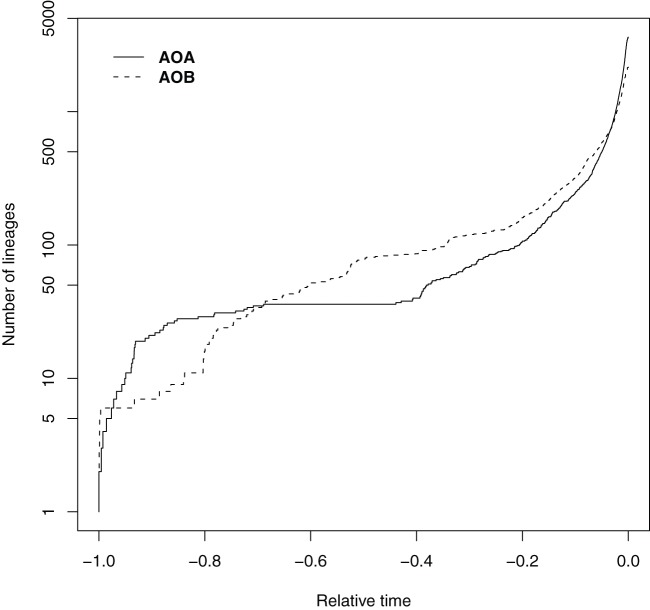
Dynamics of the cladogenesis events versus relative time using log-lineage through time plots (LTT) for the inferred phylogenies using the whole dataset of AOA and AOB. Time 0, the present.

**Figure 6:**
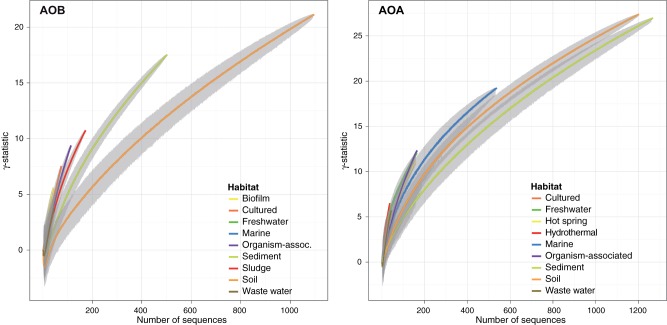
Rarefaction curves of the diversification rates (γ-statistic) for AOB and AOA in the different habitats studied.

Finally, we explored the historical context of the evolutionary and diversification processes captured in the reconstructed phylogenies. Soil ammonia oxidizers were placed on the basal position near the phylogenetic tree root both for AOB and AOA. To capture the information contained along the diversification process we represented cladogenesis events versus relative time using lineage-trough time plots (LTT, Fig. 5). A consistent increase in the net diversification rate towards present was observed with accelerated recent diversification events and high γ-statistic values for the two life domains (i.e., γ_AOB_ = 34.96, and γ_AOA_ = 45.37). The diversification dynamics in earlier times showed, however, differences between domains with AOB showing a more constant cladogenesis through time whereas AOA apparently experienced two fast diversification events apparently separated by a long steady-state episode (Fig. 5). Interestingly, for most of the habitats γ_AOA_ was higher than γ_AOB_ (Fig. 3C) and for all the cases the internal phylogeny nodes were closer to the tips than expected under a constant rate of diversification. We explored for each single habitat the historical patterns using a rarefaction analysis to correct for unequal sample size (Fig. 6). Interestingly, AOB and AOA in soil and sediment experienced earlier bursts of diversification than in the remaining environments. Afterward, soil AOB experienced a decelerated diversification rate as compared with soil AOA. In addition, AOB in sediments significantly accelerated its diversification events as compared with the soil counterparts. Finally, ammonia oxidizers from habitats usually eutrophic and rich in ammonium such as wastewater and sludge, showed accelerated diversification rates towards the present.

## Discussion

We have shown consistent differences in the phylogenetic richness among habitats and between domains, with different spatial distribution of the genetic richness (i.e., AOB were phylogenetically more interconnected whereas AOA were more phylogenetically clustered by habitat). These findings suggest differential adaptations of ammonia oxidizers to the large repertory of environmental conditions present in each habitat. In fact, Thaumarchaeota are one of the most widely distributed and abundant groups of microorganism on the planet found in all types of environments, ranging from marine and coastal environments [23], [27], neutral and acid soils [9], [28], hot springs [29], [30], remote alpine lakes [31] and slush layers in ice-covered lakes [32]. As recently shown [33], AOA have followed different evolution paths suggesting specific physiological adaptations to environments being habitat filtering, salinity and life style (soil and sediment) the main drivers of the community phylogenetic structure as captured by habitat clustering and UniFrac metrics.

Quantitative differences for the community diversity of ammonia oxidizers among different habitats were also important, and especially relevant in the case of soil ammonia oxidizers. Interestingly, the soil AOB community structure was the most dissimilar among the AOB, and for the remaining habitats AOB showed lower diversification rates and higher PSV values. This may be in agreement with the fact that different habitats for different clusters within a single AOB genus have been reported (e.g., *Nitrosospira*, [17] and references therein). AOA, in turn, showed a more heterogeneous community composition among habitats, but specific monophyletic soil groups have been reported [33]. There are also close links between pH and the relative contributions of bacteria and archaea to soil nitrification, AOA being more favoured at the lowest pH [28]. Overall, the accelerated diversification rates in soil AOA may suggest the existence of tight habitat-phylogeny associations in AOA, while in AOB these associations may be not so significant.

AOB and AOA coexist and have to compete for the same resources. In fact, the different degrees of ecological success reported for different assemblages [11], [17], [18], [28] suggest that ecological and evolutionary segregation have been acting differently in each domain along the process. AOB for instance, appeared not well adapted to develop in extreme habitats such as hot springs and hydrothermal vents, whereas AOA were apparently less favoured in hypereutrophic sites as sludge (but see, e.g., [14]). A higher substrate affinity and lower tolerance to high substrate concentrations of archaea over bacteria has been detected in one marine isolate [18] although AOA can survive in high ammonia concentrations in soil [34]. Experiments mimicking the conditions of both unfertilized soils and soils receiving moderate and high levels of inorganic fertilizer [11] showed that ammonium concentration is a more important factor modulating the community structure in AOB than in AOA. The highest ammonium concentrations were also more favorable for the growth of AOB. Overall, nitrogen concentration seems to play a major role in the AOA-AOB interactions. Interestingly, we observed in our analysis that environments permanently rich in nitrogen showed acceleration in the diversification rates that could promote the emergence of new *amoA* variants.

It has been hypothesized that archaea, in general, are better adapted to deal with chronic energetic stress [35], and this fact may also be captured by the phylogenetic community analysis and inferred population history. Thus, in AOA we observed a LTT plot with a sigmoidal behavior, i.e., two big diversification events, one on the early stage of diversification process and another one on the more recent lineages, and an apparent steady-state between them with constant diversification rate. This feature could be interpreted as an initial high rate diversification process that generated a large number of lineages. These lineages could initially have colonized all available habitats. Then, the diversification remained constant until ecological factors triggered a second diversification event. This second episode could be related to microevolutionary events [36] that may facilitate the adaptation of new lineages to new emerged environmental conditions or opportunities. It is interesting to note the high γ-statistic values and the LTT plot shapes in AOB and AOA for soil and sediment, suggesting a competitive race in these habitats. Overall, the low identity percentage at the nucleotide level and the high γ-statistic values suggest that the *amoA* gene is still under an active process of evolution.

The phylogenetic reconstruction was the critical step in the approach. The resulting trees were in agreement with other phylogenetic reconstructions found in the literature showing for instance separated AOB clusters such as *Nitrosomonas*-like and *Nitrosospira*-like, or AOA Cluster-S and Cluster-M [24]. In fact, the phylogeny of *amoA* genes is largely congruent with the picture derived by 16 S rRNA genes analysis, and therefore the habitat-phylogeny distribution patterns found for the *amoA* genes may provide strong hints for the diversity (richness and evenness) of AOB and AOA at the global scale. In addition, the habitat annotation based on the EnvO-Lite ontologies solved two important concerns for massive comparative studies. First, it reduced the number of different ecosystems to a few general habitats that could be easily translated to a globally meaningful classification. Second, it solved the need to be standard, and the ontologies in information sciences are the highest current standard [37]. Being standard means that further studies can be directly and objectively compared, and this will facilitate a comprehensive knowledge base not only for comparative studies but also for integrative database analyses. Finally, although we did an intensive search and a strict filtering process of the publicly available DNA sequences, our results and conclusions are, of course, limited by both the intrinsic biases of the information deposited in databases, and the different methodologies and experimental procedures carried out by different research teams. We cannot rule out, for instance, the inherent biases of PCR amplification and the use of specific amoA primer pairs to generate the data deposited in GenBank from the original environmental samples. To minimize these limitations, the approach taken in this study combined classical phylogeny and community phylogenetics using thousand of sequences from hundred of sites which provided statistically consistent patterns between and within domains at a global scale. These patterns may certainly generate further working hypotheses and help in setting up more accurate experimental designs to improve current knowledge of the ecology and evolution of biological ammonia oxidation.

## Methods

### Sequence collection

Two Bioperl scripts were used as wrappers to the Entrez Programming Utilities [38] to search and retrieve *amoA* sequences from NCBI GenBank database release 178 (June 2010). First, the *Esearch* utility was used to capture the *amoA* sequences that match the search string “*amo subunit A or ammonia monooxygenase subunit A or ammonia monooxygenase α subunit or Amo α subunit or amoA OR ammonia monooxygenase A or ammonia monooxygenase or ammonium monooxygenase or ammonium monooxygenase A) NOT (genome or chromosome or plasmid)*”. Next, *Efetch* retrieved the entries found by *Esearch* in a Genbank formatted flat file to get ancillary environmental information. Overall, 21,603 sequences were retrieved and stored in a postgreSQL database using the associated metadata as information source. Additionally, thaumarcheotal *amoA* sequences recently described from high mountain lakes [19], [32] and *amoA* from archaeal and bacterial complete sequenced genomes were included in the data set. Sequences were checked to validate the annotation using HMMER 3.0 [39] in combination with the PFAM 24.0 [40] models PF02461 and PF12942 for the bacterial and archaeal *amoA* domains, respectively.

### Sequence data preparation

The high quality annotated *amoA* genes dataset was built as follows. Sequences were split by domain, initially resulting in 11,738 sequences for AOA and 9,865 sequences for AOB. Sequences that lack the isolation source tag, CDS tag, those which annotated product was not an ammonia monooxygenase subunit A, and sequences that contained more than 0.1% ambiguous positions were automatically removed. Sequences were further classified by isolation source; all sources with less than five sequences were removed. We ended with 300 different isolation sources (sites), with 153 sites for AOB and 147 sites for AOA. Next, sequences for each site were clustered at 98% identity at the nucleotide level with CD-hit [41] to reduce redundant sequences. Sequences were clustered by site to keep identical sequences found in different environments. In addition a Perl sequence quality checking script was used to remove sequences considered too short, i.e, those lengths being less than two times the standard deviation of the overall sequence mean length for all sequences in each domain. The final data set contained 3,619 archaeal and 2,157 bacterial *amoA* encoding gene sequences. Final nucleotide lengths were 589±67 for AOA, and 489±88 for AOB.

### Envo-Lite annotation

The 300 isolation sources were manually annotated and reduced to 11 different habitat types using environmental ontologies, a standardized project of the of the Genomic Standards Consortium (www.environmentontology.org/). We used the *Lite* version of EnvO (former *Habitat Lite* [37]) that reduces the controlled vocabulary to 20 terms. EnvO provides a controlled and structured vocabulary with defined relationships between its terms allowing efficient and accurate software manipulation, data retrieval and integration. Sequences from laboratory microbial strains were assigned to the original habitat from which they were initially isolated whereas the Envo-lite category “cultured” (i.e., controlled habitat created by humans through laboratory techniques) only contained sequences from both artificial biofilters and bioreactors. We classified separately wastewater and sludge habitats according to the EnvO-lite definitions as follows; wastewater as liquid water that has been adversely affected in quality by anthropogenic influence, and sludge as the residual semi-solid material left from domestic or industrial processes, or wastewater treatment processes. In addition, habitat annotations that only contained a few sequences were combined in a superior hierarchical level (e.g., animal-associated and plant-associated habitats were grouped as organism-associated habitat).

### Phylogenetic analysis

The *amoA* sequences were aligned with MAFFT [42], automatically edited with GBlocks [43] and manually checked and trimmed. A custom Perl script calculated the parameters needed for Gblocks. The final alignment length was 467 positions for AOA and 351 positions for AOB. Substitution saturation in the sequences was checked after plotting distances calculated using Jukes-Cantor, Kimura and raw distances (proportion of different sites), respectively. The plots showed no saturation, so the transition/transversion ratio did not affect the estimated distances. The *amoA* phylogenetic trees from the nucleotide alignments were inferred by the MPI variant of RaxML v7.2.8 [44]. Phylogenetic inference was run using the rapid BS algorithm under the GTRCAT model and 20 maximum likelihood searches with 1000 bootstrap replicates to find the best-scoring tree under the GTRGAMMA model. The best phylogenetic tree estimated by RAxML was drawn with iTOL [45]. Environmental data sets were created and used in iTOL to graphically show the Envo-Lite annotation.

To find the level of sequence identity for each environment, a pairwise alignment all-against-all was carried out for each domain using uclust 1.4 [46].

### Community phylogenetics

Differences in phylogenetic composition of nitrifying communities among environments were analyzed with UniFrac β-diversity metric [47]. To statistically analyze the phylogenetic richness and how diversity was structured in each habitat we calculated phylogenetic diversity (PD) and phylogenetic species variability (PSV) indexes from the inferred trees [48]. PD was calculated as the sum of the branch length with 1000 randomizations to avoid the sample size effect. PSV reflects the phylogenetic relationships between taxa, being closer to 1 when all taxa are poorly related (i.e., star phylogeny) and closer to 0 when taxa are closely related. To correct for unequal sample sizes, randomized subsamples for each habitat were run [49]. We also calculated PD rarefaction curves to show how new sequences added larger branch length to the phylogenetic trees.

To graphically show relationships among habitats we used the Gephi 0.8 open source software for graph visualization and analysis (http://gephi.org/tag/0-8/) and undirected weighted networks on the UniFrac distance matrices and the pairwise alignments. In the UniFrac graph network, vertices correspond to the habitats and the weight of the edges is 1-UD, so the edges represent how similar two communities are.

To estimate divergence time, the original trees were transformed to ultrametric trees through the mean path length method (MPL) [50] as rate smoothing technique. We scaled the tree root at relative time 1, and then the tree was calibrated using the root age value. In order to visualize the events of diversification and to measure their changes among habitats we plotted lineage-through-time plots and calculated the γ-statistic [51]. For diversification events constant through time, the parameter γ equals zero and a straight line in LTT is expected. If the diversification slowed then, γ<0 and the LTT plot lays above the straight line (the tree internal nodes are closer to the root than expected under a constant rate of diversification); γ>0 indicates acceleration through time in the rate of lineages accumulation (the nodes are closer to the tips than expected). Rarefaction curves were calculated with the γ value for each habitat.

All analyses were carried out in the R environment (http://www.r-project.org/) using APE [52] and Picante [53] packages.

## Supporting Information

Table S1 AOB and AOA number of sequences (N), phylogenetic diversity (PD), phylogenetic species variability (PSV), and diversification rate (**γ** statistic) for each habitat included in the *amoA* gene phylogenetic analysis. Habitats sorted by decreasing PD values. The “cultured” habitat corresponds to engineered habitats (i.e., bioreactors and biofilters) as standardized in the EnvO-Lite annotation.
(PDF)
