# Supplementary material for: Habitat-Associated Phylogenetic Community Patterns of Microbial Ammonia Oxidizers
Source: PLoS One. 2012 Oct 9;7(10):e47330. doi: 10.1371/journal.pone.0047330 (PMC3467245; doi:10.1371/journal.pone.0047330)
Supplement: Table S1 — AOB and AOA number of sequences (N), phylogenetic diversity (PD), phylogenetic species variability (PSV), and diversification rate (γ statistic) for each habitat included in the amoA gene phylogenetic analysis. Habitats sorted by decreasing PD values. The “cultured” habitat corresponds to engineered habitats (i.e., bioreactors and biofilters) as standardized in the EnvO-Lite annotation. (PDF) [file pone.0047330.s001.pdf]

Table S1. AOB and AOA number of sequences (N), phylogenetic diversity (PD), phylogenetic species variability (PSV), and diversification rate (γ-statistic) for each habitat included in the *amoA* gene phylogenetic analysis. Habitats sorted by decreasing PD values. The "cultured" habitat corresponds to engineered habitats (e.g.., bioreactors and biofilters) as standardized in the EnvO-Lite annotation.

AOB	**N**	**PD**	**PSV**	**γ-statistic**
**Sediment**	516	7.099	0.519	17.924
**Waste water**	46	6.835	0.329	3.797
**Cultured**	75	6.764	0.515	7.536
**Organism-associated**	114	6.431	0.561	9.385
**Freshwater**	32	6.289	0.465	10.743
**Sludge**	173	6.037	0.335	2.065
**Soil**	1097	5.622	0.620	21.140
**Marine**	52	4.019	0.409	4.315
**Biofilm**	43	3.659	0.245	5.572

AOA	**N**	**PD**	**PSV**	**γ-statistic**
**Sediment**	1268	8.768	0.323	26.976
**Organism-associated**	165	8.400	0.261	12.332
**Hot spring**	158	7.801	0.567	11.103
**Soil**	1202	7.350	0.431	27.393
**Freshwater**	164	6.720	0.278	12.051
**Waste water**	45	5.782	0.459	5.685
**Marine**	538	4.587	0.138	19.240
**Cultured**	37	4.254	0.099	5.558
**Hydrothermal vent**	38	3.273	0.140	6.495
